# Pleural plaques by inhalation of asbestos fibers

**DOI:** 10.47626/1679-4435-2020-493

**Published:** 2020-12-11

**Authors:** Diemen Delgado, Oscar Ramírez, Nayab Sultan, Patricio Miranda, Ashley Delgado

**Affiliations:** 1 Universidad de Guadalajara, Instituto de Seguridad y Salud en el Trabajo - Guadalajara (JAL), México; 2 Universidad Científica del Sur, Escuela de Postgrado de Medicina Ocupacional y Medio Ambiente - Lima (LIM) Peru; 3 University of Birmingham, School of Geography, Earth and Environmental Sciences - Birmingham (BI), United Kingdom; 4 Instituto de Salud Pública de Chile, Departamento de Salud Ocupacional - Santiago (SCL), Chile; 5 Universidad Andrés Bello, Escuela de Medicina - Viña del Mar (VAL), Chile

**Keywords:** asbestos fibers, pleural plates, calcification

## Abstract

**Introduction:**

Asbestos fiber pleural plaque is characterized by lesions composed of fibrous tissue that are located in the parietal pleura. They usually appear in up to 3 to 58% of workers who were exposed to asbestos fiber, and 0.5 to 8% in the general population. The objective of this article is to present a case report of a patient whose chest x-ray showed pleural changes associated with exposure to asbestos fibers.

**Case report:**

A 49-year-old male patient, construction worker with a history of exposure to asbestos fibers, underwent a chest x-ray performed according to International Labor Organization (ILO) standards, which revealed focal pleural changes. Subsequently, the presence of pleural plaques was confirmed by computed tomography (CT) scan of the chest.

**Discussion:**

Chest x-ray with ILO technique is the basic instrument for the identification of diseases related to asbestos fiber exposure. The study should be completed with a CT scan of the chest, whose sensitivity is greater, allowing early detection of pleural abnormalities. It is essential to obtain a detailed occupational history, since it is the most reliable and practical method to measure asbestos fiber exposure.

## INTRODUCTION

Asbestos fibers are iron, sodium and magnesium hydrated silicates disposed in fine fibers. Asbestos is classified as a human carcinogen by the International Agency for Research on Cancer and by the World Health Organization.^1^ Human injuries resulting from exposure to asbestos, such as asbestosis, pleural plaques, pleural thickening, and pleural effusion, have been widely reported. Asbestos is estimated to be the cause of nearly half of deaths from occupational cancer worldwide, thus representing a constant public health problem.^2^

Due to its physicochemical properties, asbestos is provided with an insulating capacity and resistance to friction, which determines its use in industrial and agricultural processes. Furthermore, some inhabited buildings in our country and worldwide have fiber cement as their primary raw material, especially of their roof.^3^

In Chile, use of asbestos in the industry is prohibited by current legislation since 2001. However, materials with asbestos (ceiling, boilers, ducts, etc.) have been actively removed, extracted, and handled.

Other document way of exposure to this resistant material is found among relatives of workers^4^ who carried asbestos fibers to their households in their work clothes, shoes, etc., and among populations living near areas where the mineral is exploited,^5^ since the powder of its fibers, when suspended in the air, reaches many kilometers around.^6^

Asbestos fiber pleural plaque is characterized by injuries composed of fibrous tissue that are located in the parietal pleural, predominantly in the lateral and posterior intercostal regions, as well as in the mediastinal and diaphragmatic pleura.^7^

Pleural injuries are commonly bilateral, but not symmetrical, and the fibrotic tissue becomes coalesced and calcified over time.^8^ The pathophysiological process that originates asbestos pleural plaques still is unclear, but it is believed to be induced by low exposure. It is of utmost importance to recognize the presence of pleural plaques secondary to infectious processes that are typically located in pulmonary apices.

Pleural plaques usually appear in up to 3 to 58% of workers who had been exposed to asbestos fibers, and in up to 0.5 to 8% of the general U.S. population,^9^ with incidence increasing as the number of years of exposure increases.

Evidence has shown that imaging tests are useful as early detection methods in populations exposed to asbestos fiber; thus, it is extremely important to recognize some characteristic radiological findings on chest X-rays and/or chest computed axial tomography (CAT), since most cases are usually incidental radiological findings.^10^

An occupational history of asbestos fiber exposure and a chest CAT interpreted by an experienced radiographer are the cornerstones for the diagnosis of asbestos pleural plaque.

The aim of this article is to present a case report of a patient whose chest X-ray showed pleural changes associated with asbestos fiber exposure.

## ETHICAL ISSUES

The volunteer participant was informed on the aim of the study. The individual was not subjected to any intervention or intended modification. The authors committed themselves to manage information according to the regulations set forth by the Medical Code of Ethics.

## CASE REPORT

This is the report of a 49-year-old male asymptomatic patient who was a construction worker with no history of serious diseases or drug abuse. He was evaluated in a preventive health program within the scope of Law 16,744, which refers to Chilean regulations on work accidents and occupational diseases.

This assessment involves patients who had worked in places where asbestos can be found, and/or had handled materials with asbestos, but who are not currently exposed.

It is worth noting that, in Chile, mutual insurance companies are responsible to enforce Law 16,744, whose aim is to prevent and early detect harm derived from exposure to risk of occupational diseases, specifically from exposure to asbestos fibers in our case.

**Figure 1:**
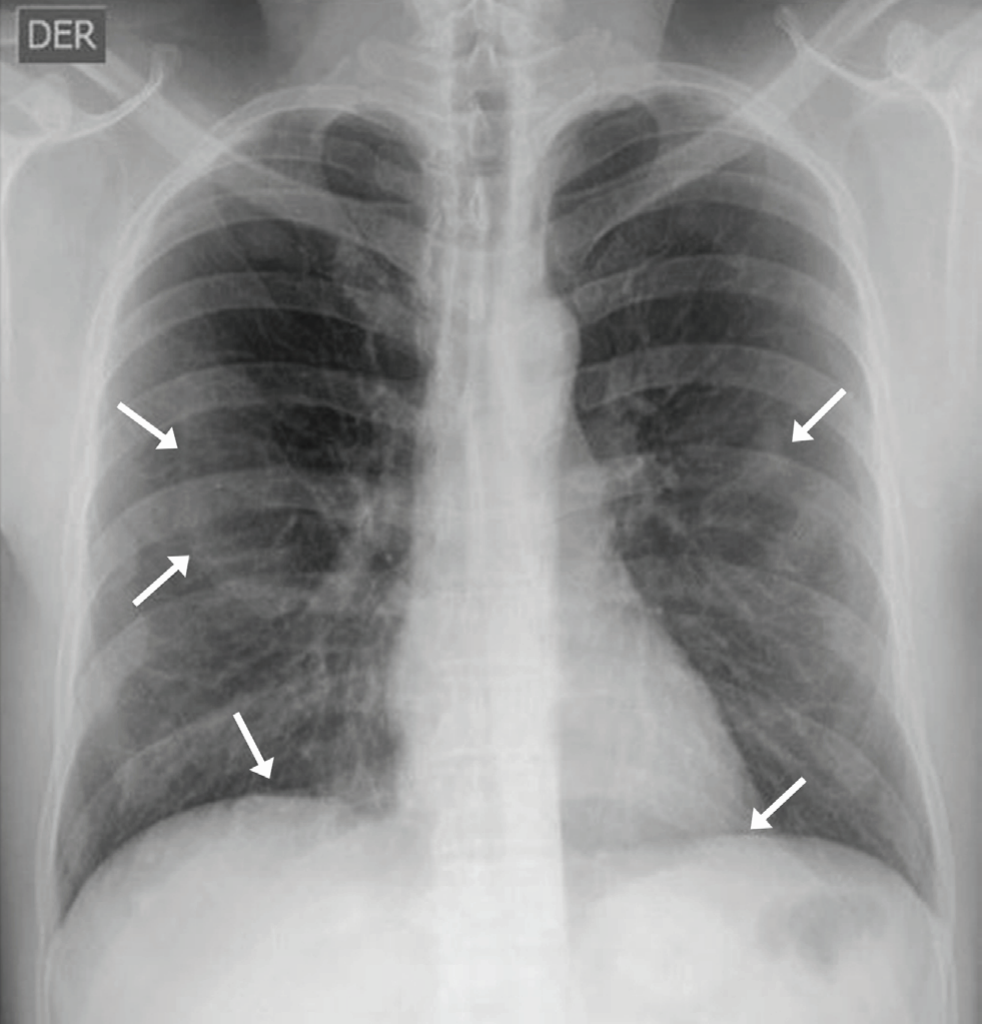
ILO chest x-ray from a patient with history of asbestos fiber exposure showing multiple pleural plaques, some of which presenting with inner calcifications.

With regard to the case reported here, the patient underwent a chest x-ray performed according to International Labor Organization (ILO) standards, which revealed focal pleural changes (Figure 1) originating from the internal surface of the ribs and extending along the adjacent intercostals muscles and at the level of the diaphragm.

**Figure 2:**
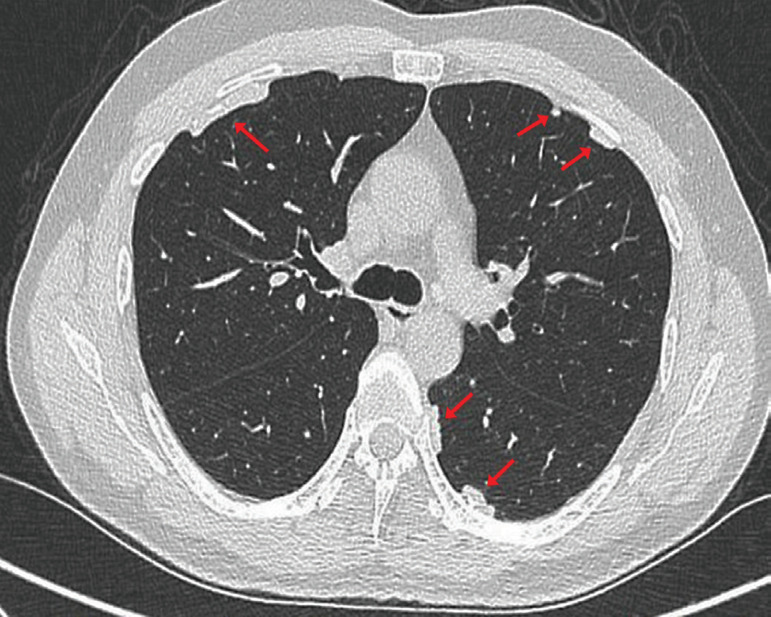
Axial section of chest computed tomography showing several calcified pleural plaques in different territories of the same patient.

**Figure 3:**
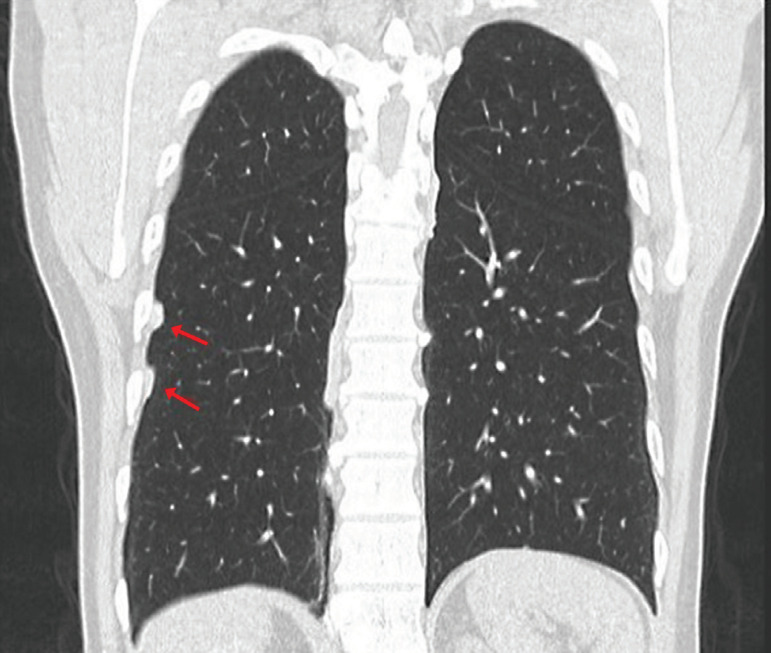
Coronal reconstruction of chest computed tomography showing at least two pleural plaques with inner calcifications on the anterolateral wall of the right hemithorax.

**Figure 4:**
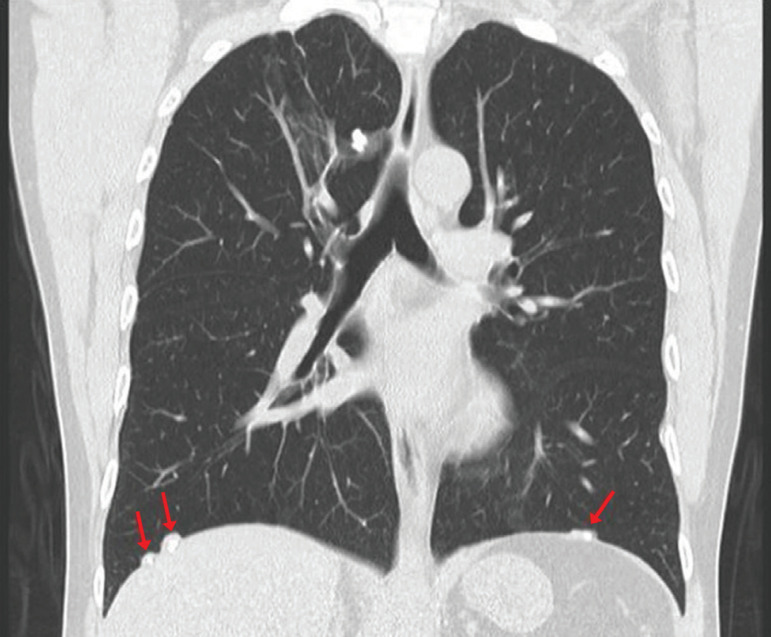
Coronal reconstruction of chest computed tomography showing at least three pleural plaques with inner calcifications. Diaphragmatic location is considered almost as a pathognomonic sign of the disease. It is worth noting that sparing of costophrenic angles supports the diagnosis of pleural plaques.

For this reason, a chest TAC was requested, because this technique provides a better definition of pleural plaques and allows for the investigation of plaques that cannot be visualized by chest x-rays, especially those located in anterior and paravertebral regions. This technique confirmed the presence of calcified pleural plaques (Figure 2, 3 and 4).

## DISCUSSION

In Chile, the production, import, distribution, selling, and use of asbestos fiber for construction has been prohibited since January 2001,^11^ while other countries have decided against total prohibition and in favor of controlled use. Currently, 90% of the global production of asbestos comes from China, India, Russia, and Brazil.^12^

The presence of asbestos pleural plaque as a chest CAT finding is still a relevant public health problem, due to the great latency between exposure and beginning of symptoms resulting from other illnesses caused by asbestos fiber exposure.

Clinically, the presence of pleural plaque does neither generate symptoms nor affect pulmonary function. ^13^ Known exposure to asbestos fiber makes us vigilant to detect other illnesses caused by this exposure that may lead to clinical impact.

Asbestos pleural plaque should be considered as a marker of exposure, and its presence may increase the risk of developing both asbestosis and mesothelioma.^14^ Asbestos pleural plaque is a slow progressing condition, and there have been no reports of development of subsequent malignancies.

However, its monitoring is recommended to serve as a marker.^15^

Currently, epidemiological information on asbestos pleural injuries in Chile is very scarce, probably due to limitations in obtaining information from those who conduct medical surveillance, and also due to difficulties in collecting death records resulting from lack of legislation on the mandatory conduction of autopsies in Chile.

Chest x-ray is a basic instrument for the identification of diseases related to exposure asbestos fiber. Its assessment is based on the 2001 ILO classification. Its limitations are lack of specificity, as well as limited sensitivity for the observation of asbestos fiber pleural disease. Hence, the study should be completed with a chest CAT, whose sensitivity is greater, allowing for early detection of pleural abnormalities.

It is essential to obtain a detailed occupational history, since it is the most reliable and practical method to measure occupational exposure to asbestos fiber, through specific questionnaires that collect not only a thorough occupational history but also information on the type of product used, duration of exposure, and the protective methods used.
